# Recent Theories of the Formation of Pus, Considered with Reference to Dental Operations

**Published:** 1887-09

**Authors:** G. V. Black


### RECENT THEORIES OF THE FORMATION OF PUS, CONSIDERED WITH REFERENCE TO DENTAL OPERATIONS.

READ BEFORE THE CONNECTICUT VALLEY DENTAL SOCIETY AT MONTREAL, P. Q., JULY 20TH, 1887.

BY G. V. BLACK, M. D., D. D. S.

The ancient and time-honored theory that the formation of pus is an act of the tissues themselves, or is a mechanical effect simply following molecular death, has, within the last few years, been questioned by the strictest experimental inquiry, and apparently has been disproven. The surgeon of the past has seen the formation of pus occur in open wounds with such promptness and regularity, that it came to be regarded as a necessary part of the series of the phenomena taking place during the processes of repair. That this formation of pus was an act of inflamed tissue, was unquestionably the opinion of the medical world down to a very recent date, and is, perhaps, yet held by the masses of the medical profession. The fluid portions of pus were regarded as liquefied portions of tissue and escaped serum, in which were commingled large numbers of so-called pus corpuscles. These latter were very properly held by almost all observers to be nothing more than ameboid cells, which had been so unfavorably placed that they had died, and were thrown off with the fluids, or had wandered out from the tissues and were carried away with the secretions. In this view of the case, pus came to be regarded generally as a normal secretion, and was looked for as an essential result in any considerable wound which involved the integrity of superficial parts.

While this was looked for as an essential occurrence, surgeons had learned to regard the characters of pus with interest in each particular case, and when this product was thick, creamy and inodorous, it was regarded as a good omen. This was called laudable pus, healthy pus, etc., and so long as this character was maintained the wound was regarded as in good condition. If, however, the pus departed from these qualities, it was known to portend evil for the patient. Therefore, different qualities of pus came to be recognized.

It had long been observed, however, that wounds in which the integrity of the skin was not disturbed, very generally healed without suppuration. A bone might be broken, and even comminuted, and if not complicated by an opening upon the surface, the wound would generally heal without the formation of pus. The case might present as much inflammatory movement as the open wound, the extent of injury might be great, and the case present a grave aspect, but yet it would very generally run through the course of repair without the formation of pus. These facts gave rise to much inquiry as to why this clinical difference, and the conjecture that it was in some way brought about by exposure to the air, was entertained.

These theories received the first severe discredit by the brilliant experiments of Joseph Lister, of Glasgow, Scotland. This surgeon, by what became known later as the Lister method, a method which had for its object the exclusions of micro-organisms from wounds, demonstrated that open wounds might be so treated that the process of repair would be essentially the same as that of internal injuries. He arrived at the idea that the formation of pus was in some way influenced by the life and growth of micro-organisms, but how, seems not to have been very clearly made out. However, the ancient idea that the formation of pus was a vital act, was not questioned by him. He seemed rather to look upon the micro-organisms as having the effect of farther depressing the vital energies of tissues already inflamed, or of increasing the inflammatory movement already present in such a way as to increase the suppuration, or to produce it in cases in which it would not have occurred without their presence. But down to the time of the World's Medical Congress, which met at London, in 1881, he certainly had no idea of questioning the general opinion that the formation of pus was a vital act of inflamed tissue. At that time Prof. Volkman, of Halle, and some others, held the opinion that without micro-organisms there was no pus formation. He says; "In the worst constitutions, and with the most disordered state of health, no suppuration takes place if septic infection is prevented." (London Congress, Vol. II, p. 362.) At that time this was regarded by Mr. Lister and others as a very extreme view.

In the consideration of this question there are certain facts that are so firmly established by direct observation that they must be regarded as fundamental, and no theory can be entertained that will not include them. What may be termed the anatomical or physical process of the formation of pus has been so well observed in the past, and agreed upon by so large a group of observers, that it must be regarded as definitely settled, and beyond the possibility of dispute. We may properly look for factors in this process heretofore overlooked, and seek different explanations of the phenomena observed, but those phenomena that have heretofore been prominently apparent are, and will ever remain, prominent phenomena of this process. Briefly, these are inflammation with its usual phenomena of heat, redness, pain, and swelling, as stated from the more superficial observation. But from the standpoint of histological inquiry, they consist essentially of certain local disturbances of the circulation, the formation within the tissues, or upon the surfaces of membranes, of a certain plastic or coagulable exudate, the gathering of white blood corpuscles, wandering cells, ameboid cells or leucocytes to the spot, and certain disturbances of the fixed cells in the focus of the inflammatory movement. These ameboid cells are properly undeveloped connective tissue cells, or cells derived from rejuvenated connective tissue, and one of their functions in the economy of nature is the repair of injuries. In this process these cells are developed into the new tissue, or tissue of repair. The plastic exudate is essential. All tissue of a primary character is first developed in a homogenous matrix. This is true of the development of the tissues of the fœtus, and is equally true of the development of tissues of repair. The plastic exudate thrown out in the process of inflammation forms the matrix in which the ameboid cells develop. These cells are always found imbedded within this exudate, and it is essential to their growth or development into tissue. In the formation of pus, this plastic exudate is observed to liquefy. The ameboid cells are found floating in the liquid mass, and are then known as pus corpuscles. Indeed, the cells under these circumstances are no longer capable of development. The matrix in which they were lodged, and in which, normally, their development should proceed, has become liquid, and is so changed in its chemical qualities that it fails to support them, and they die.

Furthermore, this plastic exudate, as I have said, is also thrown out among the fixed cells, the older elements of the tissue in which an inflammation occurs. Liquefaction may and does occur in this position also. Here we find that the ameboid cells mingle among the fixed cells of the part, and all become imbedded in a matrix of plastic exudate. In the event of suppuration the plastic exudate becomes liquid, and the tissue already divided by its presence dies, and is in part rendered fluid and floats away with the liquid material as shreds and cells, together with the ameboid cells. All of these combined, form what we know as pus.

In open wounds this process is observed to begin superficially, or upon the exposed surface. In the development of phlegmonous abscesses, it may have its beginning in the midst of the tissues. In the latter case, after its establishment, the further development of pus proceeds from the walls of the abscess, as in the open wound it proceeds from the surface. Ameboid cells that come to the surface tend to pile up in the form of living granulations, but more or less of them float away in the liquefying exudate that should form their matrix. In this way the formation of the matrix filled with fresh ameboid cells, which tend to develop into granulations, is constantly proceeding. And the liquefaction of this exudate, carrying with it the ameboid cells in the form of pus, is also constantly proceeding. If the former exceed the latter, the healing of the wound is being accomplished; but if the latter exceed the former, destruction of tissue and the widening of the breach of continuity is the result.

This much has been observed by histologists and pathologists for years past, and may be regarded as so firmly established that our views upon these points are not likely to be essentially changed by future observations.

The important questions which present themselves now are two, around which all others arrange themselves:

1st. What visible phenomena are found to be present in suppuration which had not formerly been observed and considered?

2nd. What is the cause of the liquefaction of the plastic exudate and the death of the ameboid cells?

The answer to the first of these questions must rest purely upon direct observation, and can be settled in no other than the experimental way. For many years past microbes have been observed in pus, but the idea that they were in anywise essential to pus formation was not seriously entertained until a comparatively recent date, and had not been considered. Many phenomena may be presented during the occurrence of such changes as that we are considering, that are purely accidental and not necessary to the process. Therefore, to establish that a certain accompanying phenomenon is really essential, it is necessary to show that it is always present, and that the process cannot proceed without it.

Observations in regard to the connection of microbes with the formation of pus have now been in progress for several years, and by the most astute observers of the world. These observations may be divided into two classes. First, examinations of pus with a view of determining whether or not microbes are uniformly present. Second, experimental production of pus under conditions intended to illustrate the possible connection of microbes with the process.

The finding of microbes in pus dates back many years. For a long time nothing was thought of the matter, for it was considered to be purely accidental and of no consequence. Until comparatively recent times, it was supposed that only the pus of open wounds was contaminated with these organisms, but as the means of search for these approached their present stage of perfection, many individuals asserted that microbes were present in all pus, whether it was from internal and previously unopened abscesses or not.

Formerly, before the methods of examination had reached the perfection they have acquired within the last few years, observers had often failed to find micro-organisms in pus from previously unopened abscesses, but recently this search has been more successful. I have myself examined the pus from a large number of abscesses, especially alveolar abscesses, and for several years past have found cocci in the pus in all cases. Not only this, but I have cultivated these organisms from abscesses, in broth, on gelatine, and upon agar-agar, and noted their characters. Dr. Earnst, of Boston, has also made a large number of cultivations from various classes of abscesses, with similar results. This class of experiments is but a repetition of observations by various observers in different parts of the world, separated by thousands of miles, and all give substantially the same testimony. All pus, no matter where formed, whether in closed abscesses deep within the tissues, or upon the surface, contains living micro-organisms, that will grow when placed in suitable cultivating media.

But the more decidedly practical plan of study, and one that appeals more strongly to the mind, consists in the effort to produce suppuration in experimental wounds from which micro-organisms are rigorously excluded. This class of experiment has been undertaken by a considerable number of persons. In the first of these the precautions were not successful, and micro-organisms gained entrance, vitiating the results and giving the impression that pus was produced under aseptic conditions. A very severe training was found necessary to the successful performance of this class of experiment. But as the plans were improved, success in the exclusion of microbes was attained. The results seem to have proven, that without the presence of microbes no amount of injury or irritation of the tissues will result in suppuration. These experiments have now been repeated by so many persons and varied in so many ways, and so many irritants have been employed, that the question seems no longer doubtful.

It seems unnecessary that I should now recite all, or any considerable portion, of the experimental evidence that has accumulated in the last few years. This may be found in our literature, and the more important portions have been collected in convenient form for reference by Dr. H. Knapp, of New York, in an article in the *Archives for Ophthalmology*, Vol. 15 (1886), page 24, and another in the *New York Medical Record* of Dec. 25, 1886. I have also cited some of these in an article in the *Dental Review* of March 15, 1887. In addition to these, the work of Dr. Earnst, of Boston, published in the transactions of the American Surgical Association for 1886, page 53, should not be overlooked.

As illustrating the character of this work for the benefit of those who may not have followed the literature, I may recite the plans of a few of these experiments. E. Scheuerlin placed irritants, such as croton oil, and turpentine, in very thin glass capsules or tubes, which were sealed hermetically and sterilized. These were placed under the skin of healthy animals with aseptic precautions, and allowed to remain until the wound by which they were introduced had perfectly healed. The tubes or capsules were then broken, to bring the irritant in contact with the tissues. By this plan of experiment a hard swelling was produced, but no pus was formed, except in one case, and in this the experimenter concludes that the wound had not perfectly healed. Cocci were found in its track, and in the pus that formed in the focus of the inflammation. This form of experimentation has been varied by a number of individuals, with similar results. Dr. Ruys and Dr. Knapp have made several series of experiments also of a similar nature, except that they have injected irritants into the anterior chamber of the eye (in rabbits), where both the irritant and the results of the irritation could be seen, and have satisfied themselves that it was impossible to produce suppuration in the absence of micro-organisms. I have myself made some experiments, by passing broaches rendered aseptic by heat through the apex of the roots of teeth, from which I had just removed the pulp under aseptic conditions, into the tissues beyond, and tearing them up as thoroughly as was practicable. Although inflammation was sometimes produced, there was no suppuration.

This accumulation of testimony drives us to the conclusion that micro-organisms are active agents in pus formation. The question as to how these act to produce pus, is the next important factor, and leads to the consideration of the second question, viz.: What is the cause of the liquefaction of the plastic exudate and the death of the ameboid cells?

This question had been discussed before the discovery that micro-organisms were in anywise related to the suppurative process, and the general idea of pathologists may be expressed in the phrases, "Over-stimulation of the part," "Intensity of the inflammatory process," Failure of nutrition," "Death of the cells from abnormal exposure," etc. On this point there was no very satisfactory opinion, the general idea being that those cells, unfavorably placed, either by the intensity of the inflammatory movement, by the partial cutting off of the supply of aerated blood, or by a generally impoverished condition of the system, died, and that the liquefaction of the matrix or exudate was the result of this death of the cells, or from the same cause. All of these suppositions, except the last, are probably very nearly correct, and should still have due consideration, but further study of the subject seems to show that the liquefaction of the exudate is dependent upon other agencies, and is the primary lesion looking to the formation of pus. It may be stated as an axiomatic principle, that no portion of the animal body is capable of self-destruction. The changes that take place are under the influence of life, and life is not self-destroying. One part of the body may destroy another, as is seen in the solution or absorption of the roots of the temporary teeth by the odontoclasts, or the absorption of bone by the osteoclasts, but the roots of these teeth or the bone have no part, except a passive one, in the process. One cell may destroy another in the same way, or one group may destroy another group. But in no case can a cell or any individual component of the body destroy itself. Therefore, the liquefaction of the exudate cannot be regarded as an act of the exudate, nor can the ameboid cells destroy themselves.

Any component of the body may die from want of nutritive support, or may be destroyed by traumatism; but in this event the material elements, the chemical forms, remain. These dead forms have no power in themselves to change. Long ago it was supposed, as was taught by Liebig, that organic matter left to itself fell into a state of molecular motion, in consequence of which its chemical form was changed. We have since learned, however, that all such transformations of matter are the results of life-force exhibited in certain micro-organisms, and that matter in itself, and by the powers inherent in it as matter, has no power to change, except as one element or compound is presented to another, and that in each instance the action of chemical affinity thus aroused tends to self-satisfaction and permanent quiet, until again disturbed by forces extraneous to itself. Now tissue that has died is left to itself; that power which controlled the presentation of element to element for the maintenance of its changes has ceased to act, and its chemical affinities are satisfied. Life causes chemical combinations that are peculiar to its purposes, by presenting element to element under peculiar relations or conditions. But there is no change of the chemical affinities of the elements. Dead material is, therefore, incapable of self-movement, or of change of chemical form. This is continually witnessed in various parts of the body. Blood clots resulting from the ligation of arteries, emboli, infarctions, in which considerable areas of tissue are destroyed, etc., rarely suppurate. In these cases it is seen that a tissue that simply loses vitality,—dies,—does not tend to change its chemical form except as it is acted upon by the living tissues about it, and, being shut off from the approach of microbes, does not take on degenerative changes. The cause of suppuration, when it does occur in these, will be explained later. Were it otherwise, with the forces inherent in matter as such, certainly the theory of spontaneous generation might be maintained.

That the liquefaction of the plastic exudate for the formation of pus is not an effect of inflamed tissue is, in the first instance, a matter for experimental inquiry rather than for theoretic consideration. This inquiry has been had as related, and this theoretic consideration is properly the result of that inquiry. In the years that have passed since the discovery of the yeast plant' by Schwan, in 1838, many succeeding discoveries have combined to show that all of those chemical changes that had been observed to occur in the various forms of fermentation and putrefaction, are results of the life and growth of micro-organisms, and in no case has matter been found capable of spontaneous change. Now it is found by the strictest experiment that those changes which result in the formation of pus are not due to the vital principle residing in the tissues of the animal, nor to physical agencies, but to the vital principle as exhibited in micro-organisms. It is, in fact, a species of fermentation of the plastic exudate thrown out in the act of inflammation. Strict experimental inquiry shows that this exudate is rendered fluid by a fermentative process, and is so changed in its chemical qualities that it no longer supports the ameboid cells. These cells then die, and in some of the forms of suppuration many of these are broken up and liquefied also. It had been supposed that contact with dead tissues was sufficient to destroy the vitality of the ameboid cells, but it has been found that they may wander into the dead tissues of infarcted regions, tissues destroyed by traumatism, or dead tissues placed in the body experimentally, and retain their vitality.

This, then, is the explanation of the suppurative process, and is in strict agreement with other forms of fermentation and decomposition: *Liquefaction of the plastic exudate by the operation of microbes, death of ameboid cells from the changed chemical character of their matrix*—these combined, form pus. These processes produce irritation, which extends the inflammatory movement. Hence the suppuration and destruction of tissue.

This power of micro-organisms to change gelatinous material to the liquid form is well illustrated by artificial cultivations. A considerable number of varieties, when grown in ordinary sterilized gelatine in test tubes, produce liquefaction quite rapidly, at ordinary summer temperature. One variety that I have recently been cultivating, a film-forming, or sheet coccus, will, when planted upon the surface of gelatine in a test tube, liquefy it progressively from top to bottom at the rate of about one-eighth inch per day, at a temperature of eighty degrees Fahr., and much more rapidly as the temperature is raised. Many others are found that liquefy gelatine similarly, but with less rapidity. It is this power of liquefying substances upon which they grow that gives certain micro-organisms the power to produce pus, rather than any quality of producing irritation or inflammation.* Most of them, indeed, produce irritation, also, and some of them do so in a very marked

* Note.—An organism capable of liquefying the plastic exudate, and thus producing pus, does not necessarily liquefy ordinary gelatine; neither does every organism that may liquefy gelatine in our test tubes act as a pus producer.

degree, especially the ordinary *streptococcus pyogenes,* so generally found in phlegmonous abscesses. In this respect there is, according to my own observation, great differences among the different varieties of pus-producing microbes. This has been so marked in my experience, that I am able, generally, to say in advance that certain microbes are absent or present in certain qualities of abscesses. For instance, I have never found the *streptococcus pyogenes* in a white, creamy pus, in an abscess that had arisen with little pain, or in a wound that had not taken on a decidedly inflammatory condition, etc. However, it is not now the intention to discuss the character of individual species, or varieties, of microbes that are active agents in pus formation. There are now about twelve varieties recognized, that are capable of producing pus, the greater part of which I have myself observed and cultivated. It is probable that additions will be made to this number in the near future.

To the dental profession, it is a fact of great interest that most of these fungi grow fairly well in the human saliva, and are found in it very frequently. I have recently made a considerable number of plate cultivations from the saliva of different individuals, for the purpose of determining how frequently these fungi might be found in the mouths of healthy persons. The number of cultivations have not been sufficient, however, to be satisfactory. In the examination of ten persons, I found the *staphlococcus pyogenes aureus* seven times, as determined by its form of growth in broth, upon gelatine and upon agar-agar, *staphlococcus pyogenes albus* four times, and *streptococcus pyogenes* three times. These trials were made by simply scraping the tongue, or some portion of the mucous membrane of the mouth, once with a platinum wire previously brought to a glow. These three forms are the most wide-spread and prevalent of the pyogenic fungi, and my supposition is that they would be found in a vast majority of healthy mouths if the search was carefully and persistently made. In a few cases that I have examined repeatedly, I have found the staphlococci everywhere in the mouth. In other cases I have been unable to find these at all, after repeated efforts.

The *streptococcus pyogenes* is so similar to the ordinary streptococci of the mouth, that it cannot be distinguished by microscopic examination, except by one who has had much observation of these species together and separate ; but its colony and its surface growth on gelatine afford a means of distinguishing them, after its manner of growth has once been learned.

Other pyogenic fungi than these three, I have found in the mouth only occasionally. I have also found them much less frequently in abscesses, and in pus from wounds, and must suppose that they are much less frequent in the air. It should be understood that any microbes in the air we breathe which are capable of growing in the saliva (and very large numbers grow in it), are likely to be found there from time to time. Besides these, there are certain species that are peculiar to the saliva, and are found in the mouth of almost every individual. These latter are comparatively few in number, and soon become familiar to one who spends much time in making cultivations from the saliva or mucous membranes of the mouth.

As dentists, we must consider that these pyogenic fungi are generally present in the mouth, and that every wound that we inflict is in danger of becoming infected by them. Under these circumstances, a study of the consequences of such infection becomes important to us and our patients. In the first place, the tissues upon which we operate, except it be the hard tissues of the teeth themselves, are the best supplied with blood and nerves of any in the body, and are on that account proportionately more resistant to evil influences of all kinds. This power of resistance applies to the encroachments of microbes, as effectively as to evils resulting from any other form of injury. Very few of the pyogenic fungi have, ordinarily, any considerable power to produce inflammatory conditions, and they have practically no power of mischief in any part of the body in the absence of inflammation, and necessarily the least about the mouth and face, where the resistance is greatest. When inflammation gives them the opportunity, some of the varieties cause a very rapid liquefaction of lymph deposits in an inflammatory focus, with increase of the inflammatory movement, until the growing fungi are surrounded by an almost solid wall of living ameboid cells—the so-called pyogenic membrane of older writers. This increase of the *living element* in the wall of the abscess has the effect of limiting the growth of the fungi, and in the end they are expelled, followed by the healing of the abscess.

Another reason for the abridgment of the damage is found in the weakening of the fungi from the accumulation of their own waste products. If we plant pyogenic fungi in peptonized beef broth, they will make a more or less vigorous growth, but after a time they sink to the bottom of the vessel, and all growth ceases. In case of the acid-forming fungi, we have learned by direct experiment that this cessation of growth is not on account of the exhaustion of the pabulum, but on account of the accumulation of the waste product, the acid, and that if this is neutralized by the addition of a proper amount of an alkaline base, forming a salt, answering the end of elimination of this product, growth will again proceed. This accumulation of the waste products of the pyogenic fungi occurs in the pus of abscesses, rendering it unfit for the continued growth of the fungi which produced it. The operations of the fungus thus become limited to the fresh exudates thrown in from the abscess wall. This is in turn limited as the walls become more solidly packed with ameboid cells—living matter—which is not so readily attacked.

This seems to be the explanation of the sharp rise and subsidence of suppuration, which may be said to be the common course of events when a focus of inflammation, arising from direct traumatism or other cause, is infected with pyogenic fungi alone. This is varied by two factors: first, the vital powers of the tissues; second, the activity or vital energy of the fungus. According as the one or the other is greater or less, will the course of suppuration be prolonged or abridged. If, however, the focus of inflammation be contaminated also with those species of fungi which produce active inflammatory condition, such as the cocci of erysipelas, or others of similar nature, the injury may be wide-spread and grave. Happily, such is but rarely the case in the class of lesions which we are called upon to treat.

Thus far we have considered open wounds only, or abscesses which may possibly have been infected from the surface. But microbes are found in all pus, whether the locality of its formation has had the opportunity of infection from the surface or not. The question now comes as to the possibility of infection by other channels, or what I shall designate as *indirect infection.* The ablest experimentation, perhaps, on the subject of indirect infection, has been performed by Becker and Krause. It having been observed that pus was occasionally formed in case of simple fracture of the limbs, in which the wound was in a position which rendered direct infection impossible, and yet that this pus contained the usual cocci, these gentlemen instituted experiments for the purpose of determining whether or not microbes could be conveyed to the spot by the blood streams, a theory that had been widely entertained soon after the introduction of the Lister dressings. This had led to a search of healthy tissues for micro-organisms, and the results satisfied bacteriologists that such tissues did not ordinarily contain them. Still, the question as to whether micro-organisms gaining access to the blood by accident might be so carried, was an open one. In this view of the case, the gentlemen named began their experiments by breaking the limbs of healthy rabbits, and found them to heal readily without the formation of pus. Then, after breaking the bone of the leg, the experiment was varied by injecting into a vein of the ear a fluid containing pus-forming microbes. In this case it was found that suppuration followed regularly, and that the same species of microbes injected appeared in the pus. These experiments, together with others of a like nature, established the fact that microbes may be carried by the blood to a focus of inflammation, and there set up suppuration. Dr. Knapp's experiments also show that pus-forming microbes gain entrance to the blood from wounds that are infected experimentally, for in his experiments with double operations on the eyes of rabbits (see *New York Medical Record* of December 25, 1886), one of which was infected and the other not, he occasionally found the same order of microbes used in the infection had gained access to the eye that was not infected, showing that direct infection into the blood streams is not absolutely necessary to the infection of an internal focus of inflammation. This teaches us that an internal focus of inflammation may become infected from a remote focus of suppuration upon the surface, or upon the mucous membranes. Therefore, it behooves the surgeon to examine his patient critically for minor points of suppuration before performing any important surgical operation.

It also serves to clear up another point. It is very well known that inflammation may arise in internal parts, notably in the joints, and continue for a considerable time without suppuration, but sooner or later suppuration will occur, though no direct infection has been possible. The explanation simply is that microbes have gained access to the blood through some breach, or possibly small and unimportant focus of suppuration. This class of cases may often be noted in teeth that have lost their pulps. They may go on for months together without suppurating, but finally suppuration occurs.

The time, in the course of a suppuration, at which the blood is most likely to contain microbes, is important. I do not know that there has, as yet, been any direct experimentation upon this point, but it is reasonable to suppose that after the formation of granulations, or the limitation of an abscess by the formation of a so-called pyogenic membrane, the danger of internal infections will be greatly diminished. This is also in harmony with what is known of the occurrence of septicemia and pyemia from wounds.

Such being the teaching of the facts now at our disposal, it becomes important that we inquire into the circumstances and conditions under which infection with these fungi is liable to occur in dental operations, and the circumstances and localities which favor the continued activity of the fungi after infection has occurred. The great prevalence of alveolar abscess, and the frequency with which the dentist is called upon to operate under conditions which favor the infection of the apical space, renders it the best example for the illustration of the principles taught by the facts recited. Since we have learned that we can only have alveolar abscess after infection of the apical space with pus-forming organismst, we have an incentive to the performance of aseptic operations which we never felt before. We have also learned that the enemy is in the saliva of our patients, and the facts at our disposal point to this as not only the most probable source of infection, but as surely the almost universal source—except infected instruments. It therefore follows that this source of infection should always be eliminated before a pulp chamber is opened. To do this it is necessary to put on the rubber-dam, and disinfect the tooth or teeth included, and to disinfect all instruments used in the operation. For this purpose suitable disinfectants should always be on the operating table. It may appear to some that in case a pulp chamber is already open, and infected, this precaution will be of little avail. Not so, for if the canals are already infected the first object will be to thoroughly disinfect them, and to do this the source of infection should first be eliminated. Then, and not until then, should we expect to successfully disinfect the canals. The fact that many root canals have been successfully disinfected without this precaution, only shows the power of the disinfectants employed. It does not serve to recommend such procedures, since the source of infection has become so thoroughly known. Very foul roots should be cleaned and disinfected with the rubber-dam in position, and the last of the operation should be done with freshly disinfected instruments. The cavity should then be perfectly sealed with a temporary filling of gutta-percha, or other suitable material. In no case should this filling be removed at a subsequent sitting, before the readjustment of the rubber-dam and disinfection of the included parts. In those cases in which the pulp chamber is opened for the first time, as in the removal of a living pulp, or a pulp destroyed by the operator, we should never have abscess occur; indeed, it should be impossible, except by indirect infection. This also is a matter that should always receive our attention. Unless the exigencies of the case especially demand it, we should not open a pulp chamber at a time when there is an active focus of suppuration in progress anywhere in the body of the patient. In this statement I do not include chronic discharges, though these must in every case be regarded as unfavorable.

If we have found the conditions favorable, abscess following the removal of the tooth's pulp should be impossible. We may pass our broach through the apical foramen and wound and lacerate the tissues, and possibly provoke an inflammatory movement, but if the root canal and the instrument be aseptic, abscess cannot occur. This I have tried experimentally in a sufficient number of instances to be convinced that it is practically true, as well as theoretically true. In several cases I have, in this way, aroused a considerable degree of inflammation, but in no case did abscess occur. These experiments were made with broaches cleaned by heat.

While all of this is true, it is absolutely essential that both the canal and the broach be aseptic. Few men who have not had practical experience in the cultivation of microbes can form an adequate idea of the readiness with which they may be carried into the tissues of the apical space by the broach. I have often passed a surgeon's platinum suture wire, after having brought it to a glow to completely disinfect it, into a foul root canal, and then into stiff gelatine cultivating media, four, five and six inches, and have seen the development of microbes along the track of the wire from end to end. If these organisms may be carried into a stiff gelatine in this way with a perfectly smooth platinum wire, what may we expect from a barbed broach thrust through a foul root canal into the tissue beyond ? If this does not produce abscess, it will be because the root canal happened not to contain pus-forming microbes. I have myself, as I think, produced abscess in this manner many times, and from what I know of the practice of others I am satisfied that such an accident is of very frequent occurrence. In the cleaning of contaminated canals, and all root conals to which the saliva has had access should be regarded as contaminated, no attempt should be made to reach the apex before the introduction of a reliable disinfectant. The danger of forcing microbes through, into the tissues of the apical space, is so great that they should be destroyed before the attempt to reach the end of the canal is made. Then we may risk passing the broach to the very end of the canal, and not before. The rule is simple enough. To have alveolar abscess, we must first have microbes in the tissues. If the canals have been exposed to the fluids of the mouth, we must not risk forcing its contents through the apex until it has been rendered aseptic. Then if the aseptic material is forced through, it may produce a transient inflammation, but it will not beget abscess.

What is the teaching of the facts developed in case of infection of the tissues ?

First, I may say that it is clear that there are many infections that are successfully resisted by the tissues, and the microbes eliminated without the formation of abscess. Second, where the area of the abscess is small and the general condition of the patient good, the tissues, by their own unaided powers, will expel the intruders with the pus first formed, or very soon thereafter, if proper opportunity is presented. This only requires free discharge of the pus, with care that there is not continuous reinfection. It should be stated that pus itself should be regarded in the same light as a stale cultivating medium, in which the organism which produced this condition is incapable of growing until transferred to fresh media; hence, when the central focus of the inflammation, or of the lymph deposit, is broken down, the organisms have become greatly depressed from the presence of their own products, and are readily expelled by the young connective tissue cells, which have formed themselves into a compact wall about the abscess. Therefore, the source of continuous reinfection, if such exist, should be eliminated, for this is the factor which begets chronicity. In case of simple chronic alveolar abscess, this source is the pulp canal. In this space the micro-organisms are out of reach of the tissues, and a new growth occurs, with a reinfection about the apex of the root, every time fresh pabulum has entered the root canal, and these infect any fresh lymph deposits about the apex of the root. Hence the abscess becomes chronic. Dead bone, with its Haversian canals, offers the same opportunity for growth of organisms in a harbor beyond the reach of the tissues. If, on the other hand, this bone be aseptic, it will be absorbed by the tissues, and removed. With the root of the tooth this sometimes occurs to our sorrow, but in case the root of the tooth becomes denuded of its tissues, this tendency is here largely counteracted by another, which is the tendency to the reinvestment of the root with cementum. But in order that either of these can occur, the part must be aseptic, or free from microbes. In any such cases the microbes, if present, have a wall to lie against, and are to that extent protected. The active tissue is on one side only, instead of surrounding them. Experience teaches us that in this case a chronic condition of abscess is in many instances maintained. This requires antiseptic treatment to dislodge the microbes, but in all ordinary cases of alveolar abscess, the elimination of the source of continuous reinfection from the root canal is sufficient to cure chronic alveolar abscess.

*As a rule, the microbes of pus formation are unable to maintain themselves continuously when surrounded by living tissues.*

---

In the presentation of this paper the plan which I have endeavored to follow has been: First, to present a synopsis of former views, showing the facts that have become firmly established. Second, to show the facts recently developed, and how they should modify the former views. Third, to show how these should modify our practice. In such an investigation, it will be found that no fact established by former inquiries has been lost, or even reduced in importance. It is only suppositions that must be dropped, for which freshly gained facts are substituted. The demonstrations of direct observation have been correct in the past, and this gives assurance that the developments of the observations of the present will stand the crucial test of future observers.

